# Suicide and its prevention: The urgent need in India

**DOI:** 10.4103/0019-5545.33252

**Published:** 2007

**Authors:** Lakshmi Vijaykumar

**Affiliations:** SNEHA and VHS, Chennai, India

Suicide is an important issue in the Indian context. More than one lakh (one hundred thousand) lives are lost every year to suicide in our country. In the last two decades, the suicide rate has increased from 7.9 to 10.3 per 100,000. There is a wide variation in the suicide rates within the country. The southern states of Kerala, Karnataka, Andhra Pradesh and Tamil Nadu have a suicide rate of > 15 while in the Northern States of Punjab, Uttar Pradesh, Bihar and Jammu and Kashmir, the suicide rate is < 3. This variable pattern has been stable for the last twenty years. Higher literacy, a better reporting system, lower external aggression, higher socioeconomic status and higher expectations are the possible explanations for the higher suicide rates in the southern states.

The majority of suicides (37.8%) in India are by those below the age of 30 years. The fact that 71% of suicides in India[1] are by persons below the age of 44 years imposes a huge social, emotional and economic burden on our society. The near-equal suicide rates of young men and women[2] and the consistently narrow male: female ratio of 1.4: 1 denotes that more Indian women die by suicide than their Western counterparts. Poisoning (36.6%), hanging (32.1%) and self-immolation (7.9%) were the common methods used to commit suicide.[1] Two large epidemiological verbal autopsy studies in rural Tamil Nadu reveal that the annual suicide rate is six to nine times the official rate.[3,4] If these figures are extrapolated, it suggests that there are at least half a million suicides in India every year. It is estimated that one in 60 persons in our country are affected by suicide. It includes both, those who have attempted suicide and those who have been affected by the suicide of a close family or friend. Thus, suicide is a major public and mental health problem, which demands urgent action.

Although suicide is a deeply personal and an individual act, suicidal behaviour is determined by a number of individual and social factors. Ever since Esquirol wrote that “All those who committed suicide are insane” and Durkheim proposed that suicide was an outcome of social / societal situations, the debate of individual vulnerability *vs* social stressors in the causation of suicide has divided our thoughts on suicide. Suicide is best understood as a multidimensional, multifactorial malaise. Suicide is perceived as a social problem in our country and hence, mental disorder is given equal conceptual status with family conflicts, social maladjustment etc.[5] According to the official data, the reason for suicide is not known for about 43% of suicides while illness and family problems contribute to about 44% of suicides.

Divorce, dowry, love affairs, cancellation or the inability to get married (according to the system of arranged marriages in India), illegitimate pregnancy, extra-marital affairs and such conflicts relating to the issue of marriage, play a crucial role, particularly in the suicide of women in India. A distressing feature is the frequent occurrence of suicide pacts and family suicides, which are more due to social reasons and can be viewed as a protest against archaic societal norms and expectations. In a population-based study on domestic violence, it was found that 64% had a significant correlation between domestic violence of women and suicidal ideation.[6] Domestic violence was also found to be a major risk factor for suicide in a study in Bangalore.[7] The population-based study has been done in various cities in India, however the Bangalore study is the only psychological autopsy study that focused on completed suicide and domestic violence. Poverty, unemployment, debts and educational problems are also associated with suicide. The recent spate of farmers' suicide in India has raised societal and governmental concern to address this growing tragedy.

## MENTAL DISORDERS AND SUICIDE

Mental disorders occupy a premier position in the matrix of causation of suicide. Majority of studies note that around 90% of those who die by suicide have a mental disorder.[9] The number of published reports specifically studying the psychiatric diagnoses of people who die by suicide has been relatively small (*n* = 15629). The majority (82.2%) of such reports come from Europe and North America with a mere 1.3% from developing countries.[8] Two case control studies using psychological autopsy technique have been conducted in Chennai[10] and Bangalore[7] in India. Among those who died by suicide, 88% in Chennai and 43% in Bangalore had a diagnosable mental disorder. However, diagnostic evaluations were not done in the Bangalore study.

Countless experts have found that affective disorders are the most important diagnosis related to suicide. In Chennai, 25% of completed suicides were found to be due to mood disorders. However, the suicide rate increased to 35% when suicide cases with adjustment disorder with depressed mood were also counted. The crucial and causal role of depression in suicide has limited validity in India. Even those who were depressed, were depressed for a short duration and had only mild to moderate symptomatology. The majority of cases committed suicide during their very first episode of depression and more than 60% of the depressive suicides had only mild to moderate depression.[10] Although social drinking is not a way of life in India, alcoholism plays a significant role in suicide in India. Alcohol dependence and abuse were found in 35% of suicides. Around 30-50% of male suicides were under the influence of alcohol at the time of suicide and many wives have been driven to suicide by their alcoholic husbands. Not only were there a large number of alcoholic suicides but also many had come from alcoholic families and started consumption of alcohol early in life and were heavily dependent. The odds ratio (OR) for alcoholism was 8.25 (confidence interval: CI 2.9-3.2) in Chennai[10] and 4.49 (CI 2.0-6.8) in Bangalore.[7] About 8% of suicides in India are committed by persons suffering from schizophrenia. Srinivasan and Thara found that the male to female ratio for schizophrenic suicides is more or less equal.[11] Although diagnosable mental disorders were found in 88% of suicides in the Chennai study, only 10% had ever seen a mental health professional. According to a government report, only 4.74% of suicides in the country are due to mental disorders.

Personality disorder was found in 20% of completed suicides. The OR was 9.5 (CI 2.29-84.11). Cluster B personality disorder was found in 12% of suicides. Comorbid diagnosis was found only in 30% of suicides.[10] A history of previous suicide attempt(s) increases risk of subsequent suicide. The OR for previous suicide attempts was 5.2 (CI 1.96-17.34) in Chennai and 42.62 (5.78-313.88) in Bangalore. In the Bangalore study, family history of completed suicides showed a greater risk of suicide (OR 7.69 CI 2.13-32.99) as compared to the suicidal risk indicated by the family history of attempted suicides. In the Chennai study, 12% had a family history of suicide (OR 1.33; CI 0.6-3.09) in first-degree relatives and 18% in second-degree relatives (Fisher Exact Probability test (FET) *P* = 0.001).

### Clusters of suicides

The media sometimes gives intense publicity to “suicide clusters” - a series of suicides that occur mainly among young people in a small area within a short period of time. These have a contagious effect especially when they have been glamorized, provoking imitation or “copycat suicides”. This phenomenon has been observed in India on many occasions, especially after the death of a celebrity, most often a movie star or a politician. The wide exposure given to these suicides by the media has led to suicides in a similar manner. Copying methods shown in movies are also not uncommon. This is a serious problem especially in India where film stars enjoy an iconic status and wield enormous influence especially over the young who often look up to them as role models.

The implementation of the recommendation of the Mandal Commission to reserve 27% of the positions for employment in Government created unrest in the student community and a student committed self-immolation in front of a group of people protesting against such a reservation. This was sensationalized and widely publicized by the media. There was a spate of student self-immolation (*n* = 31) around the country. These copycat suicides caused public outcry and was considered one of the reasons for the fall of the government in power at that time.[12]

### Social change

The effects of modernization, specifically in India, have led to sweeping changes in the socioeconomic, sociophilosophical and cultural arenas of people's lives, which have greatly added to the stress in life, leading to substantially higher rates of suicide.[13] In India, the high rate of suicide among young adults can be associated with greater socioeconomic stressors that have followed the liberalization of the economy and privatization leading to the loss of job security, huge disparities in incomes and the inability to meet role obligations in the new socially changed environment. The breakdown of the joint family system that had previously provided emotional support and stability is also seen as an important causal factor in suicides in India.[14]

### Religiosity

Religion acts as a protective factor both at the individual and societal levels. The often-debated question is whether the social network offered by religion is protective or whether it is the individual's faith. A study in Chennai found that the OR for lack of belief in God was 6.8 (CI 2.88-19.69).[15] Those who committed suicide had less belief in God, changed their religious affiliation and rarely visited places of worship. Eleven per cent had lost their faith in the three months prior to suicide. Gururaj *et al.* also found that lack of religious belief was a risk factor (OR 19.18, CI 4.17-10.37).[7]

### Legal issues

In India, attempted suicide is a punishable offence. Section 309 of the Indian Penal Code states that “whoever attempts to commit suicide and does any act towards the commission of such an offense shall be punished with simple imprisonment for a term which may extend to one year or with a fine or with both”.

However, the aim of the law to prevent suicide by legal methods has proved to be counter-productive. Emergency care to those who have attempted suicide is denied as many hospitals and practitioners hesitate to provide the needed treatment fearful of legal hassles. The actual data on attempted suicides becomes difficult to ascertain as many attempts are described to be accidental to avoid entanglement with police and courts.

## SUICIDE PREVENTION

The view that suicide cannot be prevented is commonly held even among health professionals. Many beliefs may explain this negative attitude. Chief among these is that suicide is a personal matter that should be left for the individual to decide. Another belief is that suicide cannot be prevented because its major determinants are social and environmental factors such as unemployment over which an individual has relatively little control. However, for the overwhelming majority who engage in suicidal behaviour, there is a probably an appropriate alternative resolution of the precipitating problems. Suicide is often a permanent solution to a temporary problem.

Mrazek and Haggerty's[16] framework classified suicide prevention intervention as universal, selective or indicated on the basis of how their target groups are defined. Universal interventions target whole populations with the aim of favorably shifting proximal or distal risk factors across the entire population. Selective interventions target subgroups whose members are not yet manifesting suicidal behaviour but exhibit risk factors that predispose them to do so in the future. Indicated interventions are designed for people already beginning to exhibit suicidal thoughts or behaviour.

## NONGOVERNMENTAL ORGANIZATIONS (NGOS)

India grapples with infectious diseases, malnutrition, infant and maternal mortality and other major health problems and hence, suicide is accorded low priority in the competition for meager resources. The mental health services are inadequate for the needs of the country. For a population of over a billion, there are only about 3,500 psychiatrists. Rapid urbanization, industrialization and emerging family systems are resulting in social upheaval and distress. The diminishing traditional support systems leave people vulnerable to suicidal behavior. Hence, there is an emerging need for external emotional support. The enormity of the problem combined with the paucity of mental health service has led to the emergence of NGOs in the field of suicide prevention.

The primary aim of these NGOs is to provide support to suicidal individuals by befriending them. Often these centers function as an entry point for those needing professional services. Apart from befriending suicidal individuals, the NGOs have also undertaken education of gatekeepers, raising awareness in the public and media and some intervention programmes. However, there are certain limitations in the activities of the NGOs. There is a wide variability in the expertise of their volunteers and in the services they provide. Quality control measures are inadequate and the majority of their endeavors are not evaluated.[17]

## NATIONAL PLAN

The World Health Organization's (WHO's) suicide prevention multisite intervention study on suicidal behaviors (SUPRE-MISS), an intervention study, has revealed that it is possible to reduce suicide mortality through brief, low-cost intervention in developing countries.

There is an urgent need to develop a national plan for suicide prevention in India. The priority areas are reducing the availability of and access to pesticide, reducing alcohol availability and consumption, promoting responsible media reporting of suicide and related issues, promoting and supporting NGOs, improving the capacity of primary care workers and specialist mental health services and providing support to those bereaved by suicide and training gatekeepers like teachers, police officers and practitioners of alternative system of medicine and faith healers. Above all, decriminalising attempted suicide is an urgent need if any suicide prevention strategy is to succeed in the prevailing system in India.

10^th^ September - World Suicide Prevention Day: The World Suicide Prevention Day was formally announced on 10^th^ September, 2003. Each year the International Association for Suicide Prevention (IASP) in collaboration with WHO uses this day to call attention to suicide as a leading cause of premature and preventable death. The theme for the year 2007 is “*Suicide Prevention—Across the Life Span*”. It calls attention to the fact that suicide occurs at all ages and that suicide prevention and intervention strategies may be adapted to meet the needs of different age groups. It is hoped that the theme will focus on vulnerable, ignored and stigmatized groups and also draw together researchers, clinicians, societies, politicians, policy makers, volunteers and survivors in a concerted action.

## CONCLUSION

Suicide is a multifaceted problem and hence suicide prevention programmes should also be multidimensional. Collaboration, coordination, cooperation and commitment are needed to develop and implement a national plan, which is cost-effective, appropriate and relevant to the needs of the community. In India, suicide prevention is more of a social and public health objective than a traditional exercise in the mental health sector. The time is ripe for mental health professionals to adopt proactive and leadership roles in suicide prevention and save the lives of thousands of young Indians.
